# C. Richard Conti and Chinese cardiology 1989–2022

**DOI:** 10.1002/clc.23905

**Published:** 2022-08-28

**Authors:** John Gordon Harold

**Affiliations:** ^1^ Department of Cardiology Cedars‐Sinai Smidt Heart Institute Los Angeles California USA

**Keywords:** C. Richard Conti, China, global leader in cardiology, Great Wall International Congress of Cardiology

## Abstract

The death of C. Richard Conti, MD, MACC in February 2022 marked the passing of a global leader in cardiology who played a pivotal role in the history of the American College of Cardiology and the College's outreach to the People's Republic of China.

The death of C. Richard Conti, MD, MACC (Figure 1) in February 2022 marked the passing of a global leader in cardiology who played a pivotal role in the history of the American College of Cardiology (ACC) and the College's outreach to the People's Republic of China.^1^

**Figure 1:**
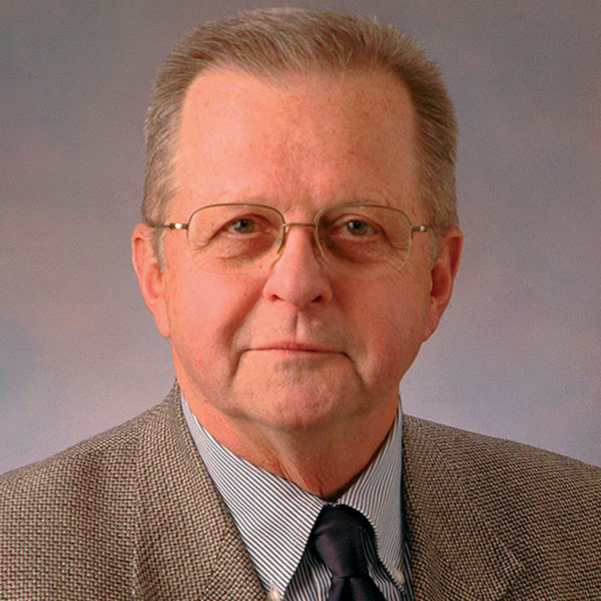
C. Richard Conti, MD, MACC

Dr. Conti served as ACC President from 1989 to 1990 and was widely acknowledged as an esteemed cardiovascular researcher, educator, and mentor.^2^ He was a pioneer in interventional cardiology and focused his research efforts on ischemic heart disease. Dr. Conti chaired the National Heart Lung and Blood Institute first national randomized trial of medical versus surgical intervention in patients with unstable angina. He was a strong advocate for advancing heart care worldwide but had a particular interest in China.^3^

Dr. Conti had received international recognition from global cardiovascular societies including the British Cardiovascular Society, French Society of Cardiology, European Society of Cardiology (ESC), College of Medicine of South Africa and the Venezuelan Society of Cardiology. Dr. Conti received the “Docteur Honoris Causa” from the Université de Marseilles in June 2000.

The ACC has a long‐standing commitment to global health dating back to its founding in 1949 by cardiologists who had fled Nazi Germany and who aspired to create an inclusive home for cardiovascular professionals.^4^, ^5^ During the 1960s, pioneered by past ACC President Eliot Corday, MD, MACC the ACC began sponsoring International Circuit Courses which rapidly expanded to involve educational events in over 40 countries. A “Medical Peace Corps” was later created under Corday's leadership modeled after the Peace Corps which were established in 1961 by President John F. Kennedy to promote world peace and friendship. Corday's vision took pre‐eminent U.S. cardiologists to less‐developed countries throughout the world with the goal of improving the quality of care worldwide. In 1973, a team of eight cardiologists representing the ACC were invited to the People's Republic of China. Deliberations between U.S. Secretary of State Henry Kissinger and Premier Zhou En‐Lai of China set the stage for the 2‐week International Circuit Courses to China, which promoted cultural exchange in the sciences. Dr. Conti continued this tradition with his educational outreach to China which began in the 1980s and continued up to the time of his untimely passing.

The ACC has developed numerous initiatives to address the global epidemic of cardiovascular disease and has functioned as an effective change‐agent on the ground working with its International Chapters, international partner societies and other stakeholders on patient and clinician education and outreach efforts targeted at prevention and population health management.^6^, ^7^, ^8^ With regards to China, these initiatives have included working with partner societies and other stakeholders to optimize patient care and outcomes through targeted education and the sharing of research and best practices.

Dr. Conti facilitated collaborative efforts to support the growth of the Great Wall International Congress of Cardiology (GW‐ICC) which celebrates its 33rd anniversary this year and is the largest scientific and educational program in China devoted to cardiovascular disease.^9^ The annual meeting of the GW‐ICC has helped to facilitate the contemporary evolution of cardiovascular medicine in China and stimulated new research initiatives including a focus on cardiovascular prevention and the prevention and treatment of noncommunicable diseases (NCDs).^10^

Dr. Conti worked closely with Professor Dayi Hu who created the GW‐ICC in 1989 and served as its Founding President from 1989 to 2014. Dr. Conti facilitated a collaboration between the ACC and GW‐ICC by creating a joint symposium at the Beijing meeting. The ACC president would lead a delegation to Beijing and discuss issues common to the Chinese and United States cardiac care teams in dealing with the global epidemic of cardiovascular disease. I was privileged to lead the ACC delegation in 2013 when I served as ACC President (Figure 2).

**Figure 2:**
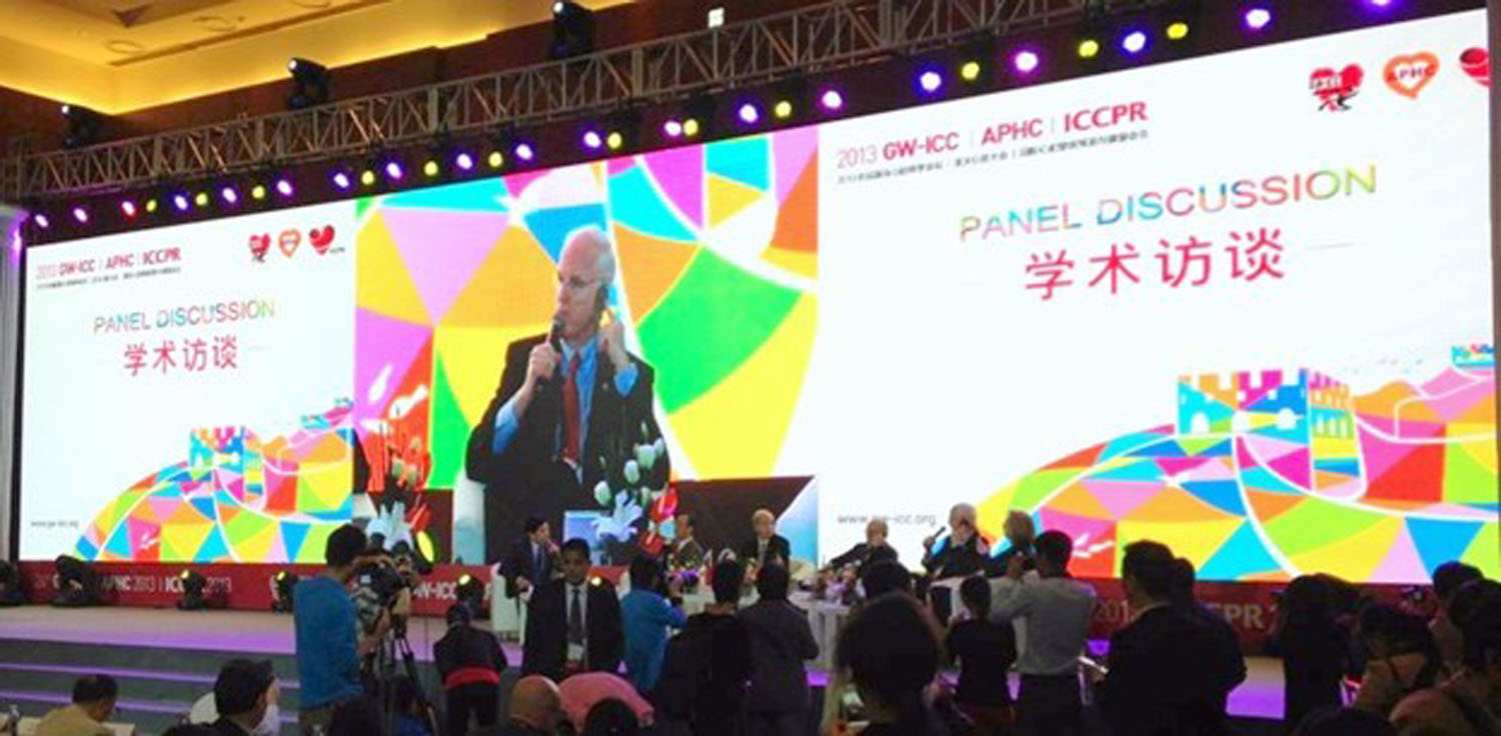
2013 Great Wall‐International Congress of Cardiology Meeting

I was honored to participate in the GW‐ICC Young Investigator session with Dr. Conti. He took special joy in chairing these sessions over the years. As part of the Young Investigators program, Chinese cardiovascular trainees would present scientific material in English and answer questions related to their presentations in English. This session was viewed as critical preparation for future presentations by these young Chinese investigators at international scientific meetings such as those held by ACC, ESC, American Heart Association, the World Heart Federation and the Asian Pacific Congress of Cardiology.

Dr. Conti actively participated in the GW‐ICC annual meetings in Beijing and their publications until the COVID‐19 pandemic and was named as honorary chair of the GW‐ICC meeting in 2005 (Figure 3).

**Figure 3:**
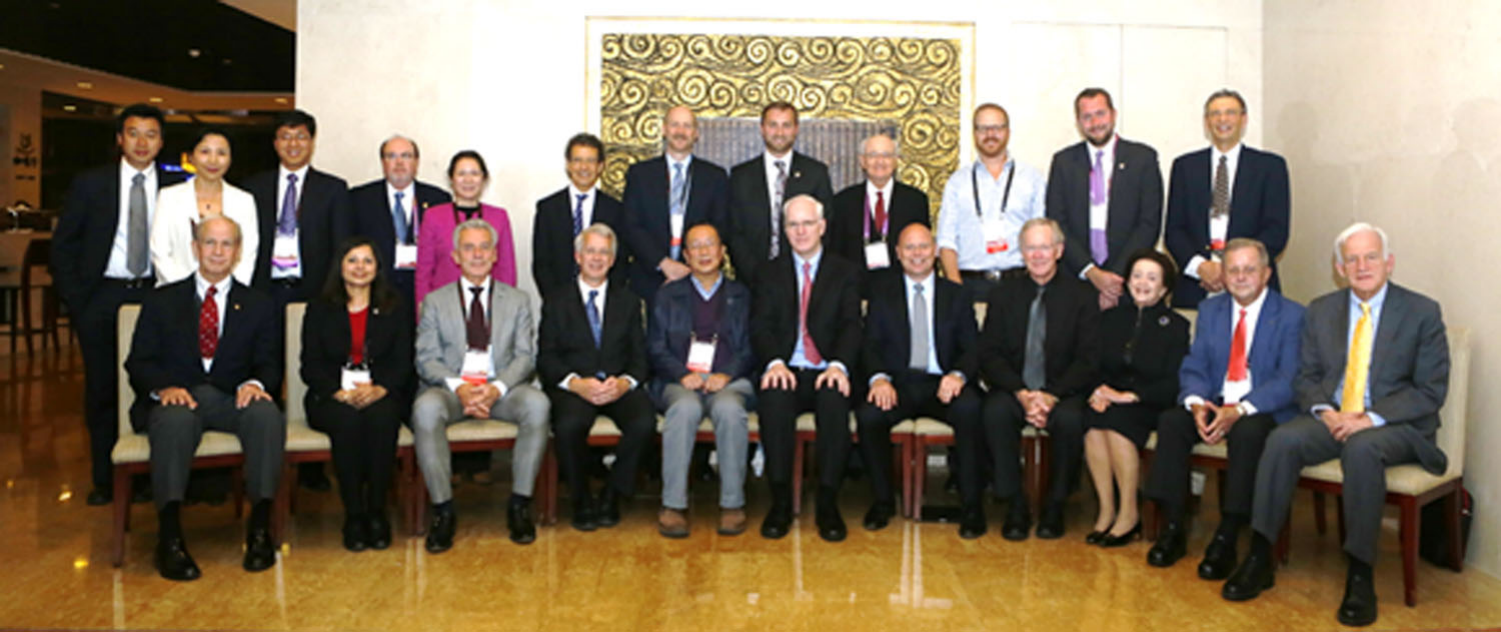
2014 Great Wall International Congress of Cardiology Meeting, Beijing, China

The GW‐ICC from 1990 promoted contacts between Chinese cardiologists and their United States and European colleagues. The GW‐ICC has also been at the forefront of the growth in Chinese cardiology research, reflecting China's growing importance in world research rankings^11^ *.* The conference has continued to introduce and promote new ideas, such as smoking cessation, exercise, rehabilitation, a health‐conscious population, and e‐health services. Dr. Dayi Hu and Dr. Conti were founding editors of the *Cardiovascular Innovations and Applications* journal which was later named as the official publication of the Great Wall International Cardiology Conference in 2016.

Dr. Conti facilitated the creation of a Chinese version of ACCEL the ACC's audio journal and the ACC China Chapter. China ACCEL was directed to young Chinese physicians so they could be more familiar with medical English. The quarterly audio journal was accompanied by printed interviews in English. As part of its missions to transform cardiovascular care and improve heart health around the world, the ACC, in collaboration with its China Chapter, the Chinese Society of Cardiology (CSC), and other stakeholders, has created multiple initiatives designed to reach Chinese physicians and patients where they live and work. ACC's China Chapter has been an exemplary facilitator in the space of patient education. Smoking and hypertension remain problematic with a high prevalence in Chinese adults. The Chapter has conducted public outreach regarding smoking cessation and hypertension management and has hosted patient education events at the GW‐ICC^12^ The Chapter also translated ACC CardioSmart patient fact sheets on various cardiovascular disease risk factors which were distributed to attendees at these events. A unique “Train the Trainer” program was implemented in 2013 focused on increasing awareness of atrial fibrillation treatment in China.^13^ Pilot centers were established throughout the country, which offered over 50 lectures and face‐to‐face exchanges in hospitals with top local and global experts in atrial fibrillation and embolic stroke prevention. The ACC also participated in the 2013 China‐USA Summit on Cardiovascular Disease Management held in conjunction with the GW‐ICC meeting (Figure 4).

**Figure 4:**
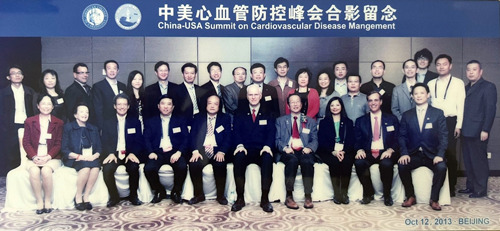
2013 China‐USA Summit on Cardiovascular Disease Management at GW‐ICC Beijing, China. GW‐ICC, Great Wall International Congress of Cardiology

In 2016, the ACC launched a cardiovascular disease education and awareness program in collaboration with the CSC to prepare physicians and hospital systems for a nationwide healthcare shift that supports heart disease prevention and optimal patient care (Figure 5).

**Figure 5:**
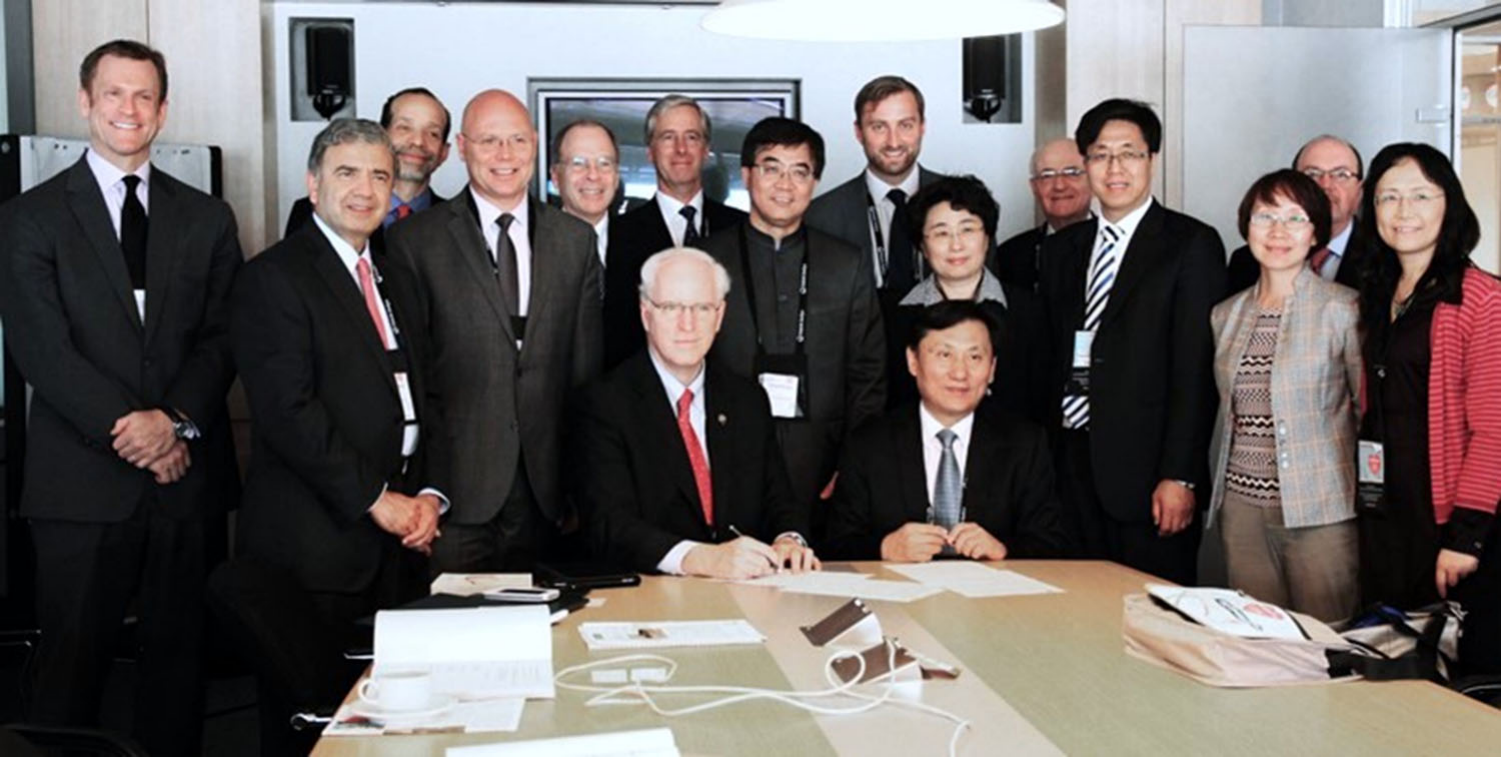
Chinese Society of Cardiology and American College of Cardiology Joint Meeting

I had the honor of participating as a consulting cardiologist under the auspices of an United Nations Educational, Scientific and Cultural Organization educational exchange program between Cedars‐Sinai Medical Center and the First Teaching Hospital at Beijing Medical University in May 1989. My Chinese counterpart was Dr. Dayi Hu who would later become the founder of the GW‐ICC. We developed a collaborative relationship which later included my interactions with Dr. Conti and the GW‐ICC which he created. The May 1989 visit of the Cedars‐Sinai delegation to Beijing coincided with several historic events including the State Visit on May 15–19, 1989, of Soviet President Mikhail Gorbachev and the concurrent student‐led demonstrations in Tiananmen Square. Our team performed coronary bypass surgery on eight male cases who ranged in age from 46 to 68 all of whom had multivessel coronary artery disease. As planned, our team acted as the principal operator on the first four patients and then the Chinese team handled the rest of the cases. In response to the increasing prevalence of coronary artery disease, the First Teaching Hospital was expanding their program in coronary bypass surgery, and this was the beginning of an enhanced educational exchange program with China.

The prevalence of coronary artery disease literally exploded in the 1990s and led to a major transformation of cardiovascular care delivery in China.^14^ Flying to Beijing in 1989 I was seated next to an American businessman whose occupation was selling tobacco paper to China for cigarette production. His unit sales were in the millions and this interaction highlighted the challenge of tobacco consumption in China and the contribution to cardiovascular risk.

China is the world's most populous country, with a population of more than 1.4 billion people. China's economic expansion since 1989 has been accompanied by a change in disease patterns from primarily infectious to NCDs, with cardiovascular disease and stroke being the primary causes of premature morbidity, mortality, and disability‐adjusted life years.^15^, ^16^ Coronary artery disease, which used to be extremely rare in China has increased considerably in prevalence over the past several decades owing to lifestyle changes, accelerated population aging and urbanization like many other industrialized nations.^17^

High blood pressure, smoking, dietary salt intake, and ambient particulate matter pollution are among the leading four risk factors contributing to Chinese cardiovascular mortality. According to the World Health Organization, these diseases are responsible for over 40% of all deaths in China and continue to result in billions of dollars in economic losses.

Reducing unhealthy lifestyle choices and improving the environment to reduce the prevalence of NCDs will be critically important as China develops healthcare policies to deal with the epidemic of cardiovascular disease. China has made significant strides in recent years diagnosing and treating patients with cardiovascular disease, though challenges remain in providing preventative care to patients at high risk of developing chronic cardiovascular conditions. Government policies including Healthy China 2020 and 2030 are designed to promote risk reduction and encourage healthier lifestyles. The impact of NCDs on cardiovascular health in China has been steadily rising, and this upward trend is expected to continue in coming years with poor diet and growing rates of obesity compounding the risks associated with continuing high rates of smoking, hypertension, diabetes mellitus and an aging population.

It has been projected that over 20 million patients with cardiovascular disease are hospitalized in China every year. The COVID‐19 pandemic has significantly impacted cardiovascular care in China and lessons learned have helped to facilitate the global management of COVID‐19.^18^ New approaches to patient workflow and delivery of healthcare were required as the pandemic further evolved. COVID‐19 had a significant impact on Chinese healthcare workers with a significant infection and mortality rate.^19^ The concept of critical care cardiology has evolved in China and given the physician shortages, physicians from other subspecialities were called on to provide patient care. In China, from January 3, 2020, to July 1, 2022, there have been 4 769 367 confirmed cases of COVID‐19 and the challenges to cardiovascular care and chronic disease management continue.

Following the lead of scientist and writer René Dubos who stated we need to “think globally, act locally,” the ACC is committed to reaching clinicians where they live and practice all around the world. Inspired by the work of Dr. Conti in China the College has continued its international outreach to this populous nation. Only by working together will we be able to achieve the College's vision of a world where innovation and knowledge optimize cardiovascular care and outcomes, as well as the broader global goal of reducing deaths from cardiovascular disease and other NCDs.^20^, ^21^

## Data Availability

Data sharing not applicable to this article as no datasets were generated or analyzed during the current study.
